# Mesenchymal stem cells derived from different perinatal tissues donated by same donors manifest variant performance on the acute liver failure model in mouse

**DOI:** 10.1186/s13287-022-02909-w

**Published:** 2022-06-03

**Authors:** Shanshan Li, Junfeng Wang, Bin Jiang, Jiang Jiang, Lilin Luo, Bingrong Zheng, Wei Si

**Affiliations:** 1 School of Medicine, Yunnan University, Kunming, 650000 Yunnan China; 2 State Key Laboratory of Primate Biomedical Research, Institute of Primate Translational Medicine, Kunming University of Science and Technology, Kunming, 650000 China; 3 Department of Hepatic and Bile Duct Surgery, The First People’s Hospital of Yunnan Province, Kunming, 650000 China; 4 Department of Obstetrics, The First People’s Hospital of Yunnan Province, Kunming, 650000 China; 5 Department of Pathology, The First People’s Hospital of Yunnan Province, Kunming, 650000 China

**Keywords:** Acute liver failure (ALF), MSCs, CL-MSCs, CPJ-MSCs, FP-MSCs, Heterogeneity

## Abstract

**Background:**

Mesenchymal stem cells (MSCs) derived from different tissues have variant biological characteristics, which may induce different performances in the treatment of diseases. At present, it is difficult to know which type of MSC is most suitable for acute liver failure (ALF), and there is no parallel study to compare MSCs from different tissues of the same donor.

**Methods:**

In this study, we derived MSCs from three different perinatal tissues of the same donor: cord lining (CL), cord–placenta junction (CPJ) and fetal placenta (FP), respectively, for compared gene expression profiles by transcriptome sequencing, and ability of proliferation and immune regulation in vitro*.* In addition, the therapeutic effects (e.g., survival rate, histological evaluation, biochemical analysis) of CL-MSCs, FP-MSCs and CPJ-MSCs on ALF mouse model were compared.

**Results:**

The transcriptome analysis showed that FP-MSCs have significantly high expression of chemokines compared to CPJ-MSCs and CL-MSCs, similar to the q-PCR result. Of note, we found that CPJ-MSCs and FP-MSCs could improve the survival rate of mice with ALF induced by carbon tetrachloride, but CL-MSCs had no difference with Sham group. Moreover, we also found that biomarkers of ALF (e.g., MDA, SOD and GSH-px) significantly improved post-CPJ-MSCs and FP-MSCs treatment, but not CL-MSCs and Sham group. However, CL-MSCs treatment leads to inflammatory reaction in the early stage (day 3) of ALF treatment but not found with other groups.

**Conclusions:**

It is important to select the MSCs derived from different tissues with variant performance for therapeutic purpose, and the CPJ-MSCs and FP-MSCs cells can significantly improve the syndrome of ALF which is highly recommended for a potential therapeutic options for ALF.

**Supplementary Information:**

The online version contains supplementary material available at 10.1186/s13287-022-02909-w.

## Background

Acute liver failure (ALF) occurs rapidly by virus or drug and may develop to a life-threatening disease when a health liver for transplantation may be the last cure [1]. Clinical manifestations usually include liver dysfunction, abnormal liver biochemical values, coagulation dysfunction, encephalopathy with multiple organ failure [2, 3]. Although orthotopic liver transplantation is the most effective treatment, the clinical application is highly limited by the shortage of donated organs, costs and lifelong administration of immunosuppression [4, 5]. Therefore, alternative treatment and regeneration strategies are urgently needed for acute liver failure [6].

Mesenchymal stem cells (MSCs) are multipotent stem cells with the abilities of self-renew, differentiation to multiple cell types and immunomodulatory [7]. MSCs isolated from perinatal tissues including cord blood [8], cord tissue [9], placenta [10] and amniotic fluid [11] have gradually been widely used in preclinical study and clinical trials due to their particular advantages on noninvasive tissue retrieval process, convenience and abundant availability. Furthermore, perinatal tissues-derived stem cells are more primitive than adult-derived stem cells [12, 13].

Although MSCs from different tissues display similar characteristics [14], differences exist among these various sources of MSCs. For example, MSCs derived from human umbilical cord and placenta show significant differences in colony forming efficiency and differentiation ability [15]. Previous study also implied that MSCs derived from umbilical cord and placenta may be suitable for various adaptation diseases because they secrete different cytokines and growth factors [16]. MSCs derived from multiple sources including bone marrow [17], adipose tissue [18], umbilical cord (UC) [19] and placenta [20] have been reported to treat ALF. However, it is hardly possible to draw the conclusion that which type of MSCs is the most suitable for ALF at present since there is no study parallelly compared MSCs derived from different tissues but based on same donors so far. Therefore, the aim of the present study is to derive various MSCs from three discrete anatomical regions of perinatal tissues including cord lining (CL), cord–placenta junction (CPJ) and fetal placenta (FP) based on same donors and parallelly compare their therapeutic effects on liver failure mouse models. All of the MSCs (CL-MSCs, CPJ-MSCs and FP-MSCs) were cultured and processed under the same protocol and conducted the following experiments: Firstly, we explored the transcriptomic profile of the three types of MSCs. Secondly, under the stimulation of inflammatory environment in vitro, the secreted factors were analyzed and the paracrine effects of three MSCs were compared. Finally, we compared the therapeutic effects on liver failure mouse models induced by carbon tetrachloride (CCl_4_) evaluated by survival rate, pathology and biochemical indexes. Our study may help to identify a potential MSC type for the treatment of human acute liver failure in future.

## Methods

### Isolation, culture and identification of MSCs

#### Cell isolation and culture

**Fig. 1:**
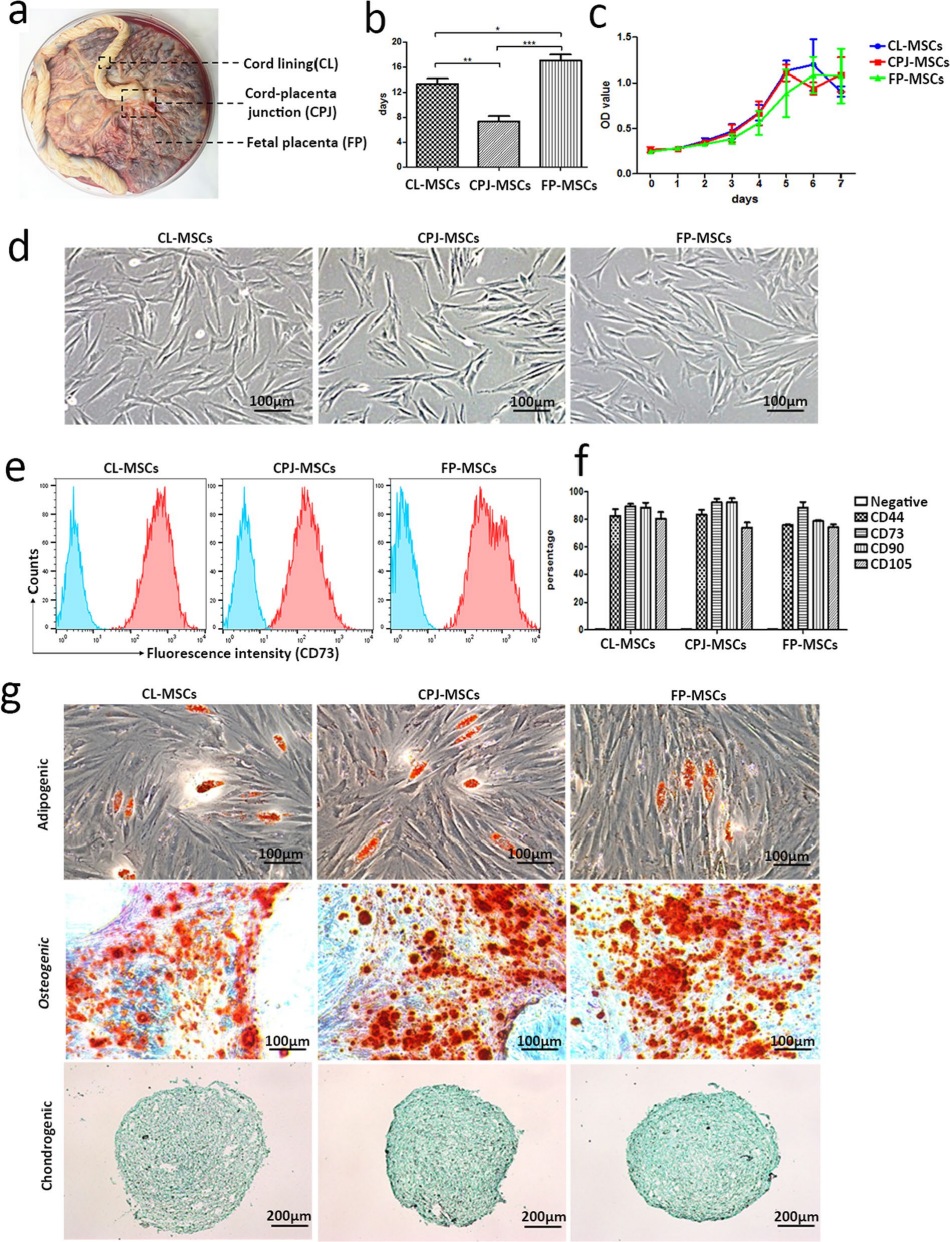
Morphology, differentiation potential and immunophenotyping profiles of CL-, CPJ- and FP-MSCs derived from same donors.** a** Schematic of the isolation of cells from the cord lining of human umbilical cord (CL), cord–placenta junction (CPJ) and fetal placenta (FP). **b** Time required for the cell density of perinatal tissue culture reached 70% (P0). **c** Cell growth curve (P3). **d** Fibroblast-like morphology of MSCs at passage 3. **e** Surface marker expression of three types of MSCs analyzed by flow cytometry (CD73). **f** Quantitative profile of surface markers expression. **g** Adipogenic differentiation (Oil Red O staining); osteogenic differentiation (Alizarin Red staining); and chondrogenic differentiation (Alcian Blue staining). * represents a significant difference (*p* < 0.05). ** represents a significant difference (*p* < 0.01). *** represents a significant difference (*p* < 0.001), *n* = 3 for each type of MSCs

Usage of human sample (*n* = 3, from healthy donors) and the protocol used in this study are approved by Ethics Committee of Yunnan first people's Hospital (KHLL2019-KY052). MSCs were acquired through direct explanted method using umbilical cords and fetal placenta donated by healthy donor immediately after giving birth. All the donors (*n* = 3) provided informed consents prior to tissue donation. Three different anatomical regions of the perinatal tissue were manually dissected including CL, CPJ and FP [15], and the explants were further cultured for deriving MSCs in vitro (Fig. 1a). Each tissue sample of the specific regions was minced and cultured in the 150-cm^2^ plates at 37 ˚C. All tissue was cultured with the DMEM medium supplemented with 12% fetal bovine serum and 1% penicillin/ streptomycin. Cellular morphologies of the CL-MSCs, CPJ-MSCs and FP-MSCs were examined and photographed using a microscope (Leica, Germany).

#### Characterization of the surface markers of MSCs

The identification of MSCs is conducted by the surface marker characterization with BD MSCs identification Kit (BD Bioscience) by flow cytometry. Briefly, the MSCs were dissociated with 0.25% trypsin and rinsed with 1 × DPBS two times. Afterward, the cells were adjusted to 1 × 10^6^ cells/mL per tube and incubated with each antibody as manual book on ice for 30 min, the antibodies including CD44, CD90, CD73 and CD105 and negative cocktail (including CD45, CD34, CD11b, CD19 and HLA-DR). The isotype control antibody cocktail was used as a negative control. The cells were rinsed with 1X DPBS twice and processed the measurement with FACS Calibur system (BD Bioscience, USA). The data were further analyzed with FlowJo software (Ver. 10).

#### Cell differentiation

The MSCs derived from each region of cord–placenta tissue were tested for the capability of tri-lineage differentiation to adipocytes, osteocytes and chondrocytes with the commercial differentiation kits (R&D Systems, USA) according to the manufacturer’s manual. Briefly, for the adipogenic differentiation, the MSCs were plated in the 6-well plate with 1 × 10^6^ cells/well, and the adipogenic differentiation media were changed every other day for 21 days. The cells were fixed with 4% paraformaldehyde (PFA) for 30 min at 4 °C. Then, the cells were assessed by staining the cells with 0.5% Oil Red O solution [21]. For the osteogenic differentiation, MSCs administration, osteogenic differentiation medium change, culture days and fixation were the same with adipogenic differentiation, and the mineralization of differentiated cells was assessed by staining with Alizarin red [21]. For chondrogenic differentiation, 2 × 10^6^ MSCs were centrifuged at 1000 rpm to form the cell pellets, and then, the cell pellets were cultured in chondrogenic differentiation medium for 21 days, then fixed in 4% PFA and embedded in OCT (Sakura, Japan) for slicing with sliced 7 microns thick with cryostat (Leica, Germany). Afterward, the sections were stained with Toluidine Blue [21]. All dyes were purchased from Sigma-Aldrich.

#### Cell proliferation assay

When MSCs were cultured to the third generation, cell counts were performed using the Cell Proliferation Reagent WST-1 (Beyotime, China) at 0–7 days to obtain data to plot a growth curve. It can be quantified using a spectrophotometer at a wavelength of 460 nm.

### Comparison of expression profiles of MSCs

#### Library preparation and transcriptome sequencing

RNA from CL-MSCs (*n* = 3), CPJ-MSCs (*n* = 3) and FP-MSCs (*n* = 3) were extracted. Sequencing libraries were generated using NEBNext® UltraTM RNA Library Prep Kit for Illumina® (NEB, USA) following manufacturer’s recommendations, and index codes were added to attribute sequences to each sample. The library preparations were sequenced on an Illumina Novaseq platform, and 150 bp paired-end reads were generated. We use HISAT2 v2.0.5 to compare the paired-end clean reads with the reference genome, and feature Counts v1.5.0-p3 was used to count the reads numbers mapped to each gene. And then FPKM of each gene was calculated based on the length of the gene and reads count mapped to this gene.

#### Differential expression gene (DEG) analysis

DEG analysis was performed using the DESeq2 R package (1.16.1). DESeq2 provides statistical routines for determining differential expression in digital gene expression data using a model based on the negative binomial distribution. The resulting *p* values were adjusted using the Benjamini and Hochberg’s approach for controlling the false discovery rate. Genes with an adjusted *p* value < 0.05 found by DESeq2 were assigned as differentially expressed.

#### Gene ontology (GO) and enrichment analysis

*GO* enrichment analysis of *DEG* was implemented by the cluster Profiler R package, in which gene length bias was corrected. GO terms with corrected *p* < 0.05 were considered significantly enriched by differentially expressed genes.

#### Data availability

The obtained transcriptome data have been uploaded into the NCBI SRA database and are accessible via the accession number PRJNA836369.

#### Gene expression analysis by qRT-PCR

For the qRT-PCR, total RNA was extracted from each MSCs using Trizol® reagent (Thermo Fisher Scientific, USA). The collected RNA was dissolved in sterile diethyl pyrocarbonate-treated water (Sangon Biotech, China), and cDNA was synthesized using a PrimeScript RT reagent kit (Takara, Japan) at 37˚C for 5 min and 85˚C for 5 s. Quantification of specific genes was performed using SYBR® Premix Ex TaqTM II kit (Takara, Japan) and CXF real-time PCR system (Bio-Rad Laboratories, USA) at particular condition (Stage 1: 95˚C for 3 s; Stage 2: 95˚C for 3 s; 60˚C for 30 s, and repeat for 37 cycles). All experiments were performed in triplicate, and the data were analyzed using the 2^−∆∆Ct^ method. All the primers were commercially synthesized (Sangon Biotech, China), and the primers’ sequence is shown in Table 1.

**Table 1:** Primers used for real-time quantitative RT-PCR

Gene		Sequence (5′–3′)	Accession
GAPDH	For	caacgtgtcagtggtggacctg	NM_002046.7
	Rev	gtgtcgctgttgaagtcagaggag	
IDO1	For	ctgcctgatctcatagagtctg	NM_002164.6
	Rev	ttgtggtctgtgagatgatcaa	
CCL2	For	agaatcaccagcagcaagtgtcc	NM_002982.4
	Rev	ttgcttgtccaggtggtccatg	
CXCL10	For	ctctctctagaactgtacgctg	NM_001565.4
	Rev	attcagacatctcttctcaccc	
IL6	For	cactggtcttttggagtttgag	NM_000600.5
	Rev	ggacttttgtactcatctgcac	
PDL1	For	gctgcactaattgtctattggg	NM_014143.4
	Rev	cacagtaattcgcttgtagtcg	
CXCL1	For	tcaccccaagaacatccaaa	NM_001511.4
	Rev	tcactgttcagcatcttttcga	
IL1B	For	gttccctgcccacagacct	NM_000576.3
	Rev	gaagacaaatcgcttttccatc	
E2F1	For	cgcatctatgacatcaccaacg	NM_005225.3
	Rev	gggtctcaggaggggcttt	
ORC1	For	aagcccagaatgaagcgacc	NM_001190818.2
	Rev	ctgaggagcggcacaacg	
CDK1	For	tggggtcagctcgttactca	NM_001786.5
	Rev	tgctaggcttcctggtttcc	
BUB1B	For	aaaccacatcctaagcaccaga	NM_001211.6
	Rev	gccaaatgttctccgcaag	

### Comparison of paracrine effects of MSCs in vitro

The MSCs (10^5^ cells) were loaded in a 6-well plate overnight with/without 20 ng/mL interferon γ (IFNγ) treatment. For the RT-PCR, total RNA was extracted from the IFNγ treated and non-treated MSCs using Trizol® reagent (Thermo Fisher Scientific, USA) and cDNA was generated using the PrimeScript RT Reagent kit (Clontech, USA). PCR reactions were carried out using the Quick-Load R Taq 2 × Master Mix (NEB, UK). The primers including GADPH, Indoleamine 2,3-dioxygenase 1 (IDO1), C–C motif chemokine 2 (CCL2), C-X-C motif chemokine 10 (CXCL10), interleukin-6 (IL6) and programmed cell death 1 ligand 1 (PDL1) were commercially synthesized (Sangon Biotech, China), and the sequences are listed in Table 1.

### Generation of ALF Mouse model and MSC infusion

#### Generation of ALF mouse model

All animal work was compliant with the protocol of Institutional Animal Care and Use Committees (IACUC) at Kunming University of Technology (PZWH-K2019-0007). Briefly, ICR mice (body weight 25–30 g) were intraperitoneally injected with 4 ml/kg CCl_4_ (Sigma-Aldrich, USA) mixed with olive oil (Sigma-Aldrich) (1:1 ratio). The acute liver failure was confirmed by histology analysis 24 h post-CCl_4_ injection. All the mice were grouped as follows: (1) control group (*n* = 15): no treatment of CCl_4_ injection or cells; (2) Sham group (*n* = 45): liver failure induction + tail vein injection of 200 μL normal saline; (3) CL group (*n* = 45): liver failure induction + tail vein injection of 1 × 10^6^ CL-MSCs suspended in 200 μL saline; (4) CPJ group (*n* = 45): liver failure induction + tail vein injection of 1 × 10^6^ CPJ-MSCs suspended in 200 μL saline; and (5) FP group: liver failure induction + tail vein injection of 1 × 10^6^ FP-MSCs suspended in 200 μL saline (Table 2). When MSCs were cultured to the sixth generation, MSCs transplantation was performed 24 h after CCl_4_ injection. CL-, CPJ- and FP-derived MSCs from the three donors were injected into ALF mice from CL group, CPJ group and FP group, respectively. For each type of MSCs from each donor, 15 mice were infused. Each group involved in 45 mice.

**Table 2:** A tabular summary of study groups

Group	Liver failure procedure	Treatment	Animal number
Control	Normal saline	Normal saline	N = 15
Sham	CCL_4_	Normal saline	N = 45
CL	CCL_4_	CL-MSCs	N = 45
CPJ	CCL_4_	CPJ-MSCs	N = 45
FP	CCL_4_	FP-MSCs	N = 45

#### Long-term survival

The mice survived more than 21 d post-CCl_4_ injection were considered as survivors. ICR mice were randomized into four groups (*n* = 45 per group) and were intravenously injected with CL-MSCs, CPJ-MSCs, FP-MSCs and saline corresponding to CL, CPJ, FP and Sham groups, respectively. The survival rate was calculated by the log-rank test.

#### Histological evaluation

Liver samples were harvested at the time points indicated above (24 h, 3 d, 7 d, 21 d post-cell transplantation). The tissues were fixed in 4% paraformaldehyde and then were embedded in paraffin for slicing. The liver sections (7 μm thickness) were subjected to hematoxylin and eosin (H&E) staining. The sections were observed under inverted microscope (Olympus, Japan).

#### Biochemical analysis of serum and liver homogenate

Blood serum was separated by centrifugation at 3,000 rpm for 15 min within 1 h after blood collection and stored at − 80 °C until analysis. Aspartate aminotransferase (AST) and alanine aminotransferase (ALT) were measured using an automatic biochemical analyzer (Roche, Switzerland). Liver sample was collected at different time points (24 h, 3 d, 7 d and 21 d), and 1 × PBS containing 1% PMSF was added. The sample was chopped and homogenized by a tissue grinder and followed a centrifugation at the speed of 2000–3000 rpm for 20 min. The supernatant was collected to detect superoxide dismutase (SOD), glutathione peroxidase (GSH-px), malondialdehyde (MDA) and IL6. Briefly, MDA is a product of lipid peroxide which is associated with liver disease (e.g., hepatitis and ALF) when a large amount of hepatocyte damaged [22]. SOD is the main scavenger of free radicals in vivo. It can remove the highly active superoxide anion produced in the process of lipid peroxidation. When the liver damage/failure happens, the content of SOD in cytoplasm will decrease [23]. GSH-px is the major component of enzymatic protective system for anti-lipid peroxidation, and it usually decreases when hepatocytes get damage/failure [24].

### Statistical analysis

Student’s t test or one-way ANOVA was employed for statistical comparisons (Spss16.0). The results of the bioinformatics analysis were visualized using GraphPad Prism 5 software. The statistical significances were calculated as *p* values, and *p* < 0.05 was considered as statistical significance. Student’s t test was used for comparison between two groups and one-way ANOVA for comparison among three and more groups. All data are expressed as means ± SEM.

## Results

### Primary culture and identification of CL-MSCs, CPJ-MSCs and FP-MSCs

The MSCs may derive from the different parts (i.e., CL, CPJ, FP) of human perinatal tissues in vitro with the same protocol and reagents (Fig. 1a). The tissues of CL, CPJ and FP were cultured in cell culture dishes. The cell density of the three kinds of MSCs reached 70% at 12–15 days, 6–9 days and 15–18 days, respectively (Fig. 1b). The growth curves of three kinds of MSCs are shown in Fig. 1c. CL-MSCs, CPJ-MSCs and FP-MSCs maximize proliferation on the sixth day, fifth day and seventh day (Fig. 1c). The morphology, immunophenotype profiles and tri-lineage differentiation potential of CL-MSCs, CPJ-MSCs and FP-MSCs were examined at passage 3. During the primary culture, CL-MSCs, CPJ-MSCs and FP-MSCs adhered to the plastic dishes in a scattered manner and exhibited similar morphology to each other, and the cells appeared fibroblast-like, elongated and spindle shape with single nucleus (Fig. 1d).

The results of immunophenotype profiles analyzed by flow cytometry showed that either CL-MSCs, CPJ-MSCs or FP-MSCs expressed high levels of the positive markers CD44, CD73, CD90 and CD105, but did not express the negative cocktail markers (Fig. 1e, f and Additional file 1: Fig. s1).

The CL-MSCs, CPJ-MSCs and FP-MSCs could differentiate into adipocytes, osteocytes and chondrocytes in vitro (Fig. 1g). After the adipogenic differentiation, numerous neutral lipid droplets stained with Oil Red O were observed in the cytoplasm of CL-MSCs, CPJ-MSCs and FP-MSCs. After the osteogenic differentiation, the cells presented an aggregation of micronodules or calcium deposits that were stained by Alizarin Red. The chondrogenic differentiation of all types of MSCs was confirmed by Alcian Blue stain.

### Analysis of DEGs of CL-MSCs, CPJ-MSCs and FP-MSCs

**Fig. 2:**
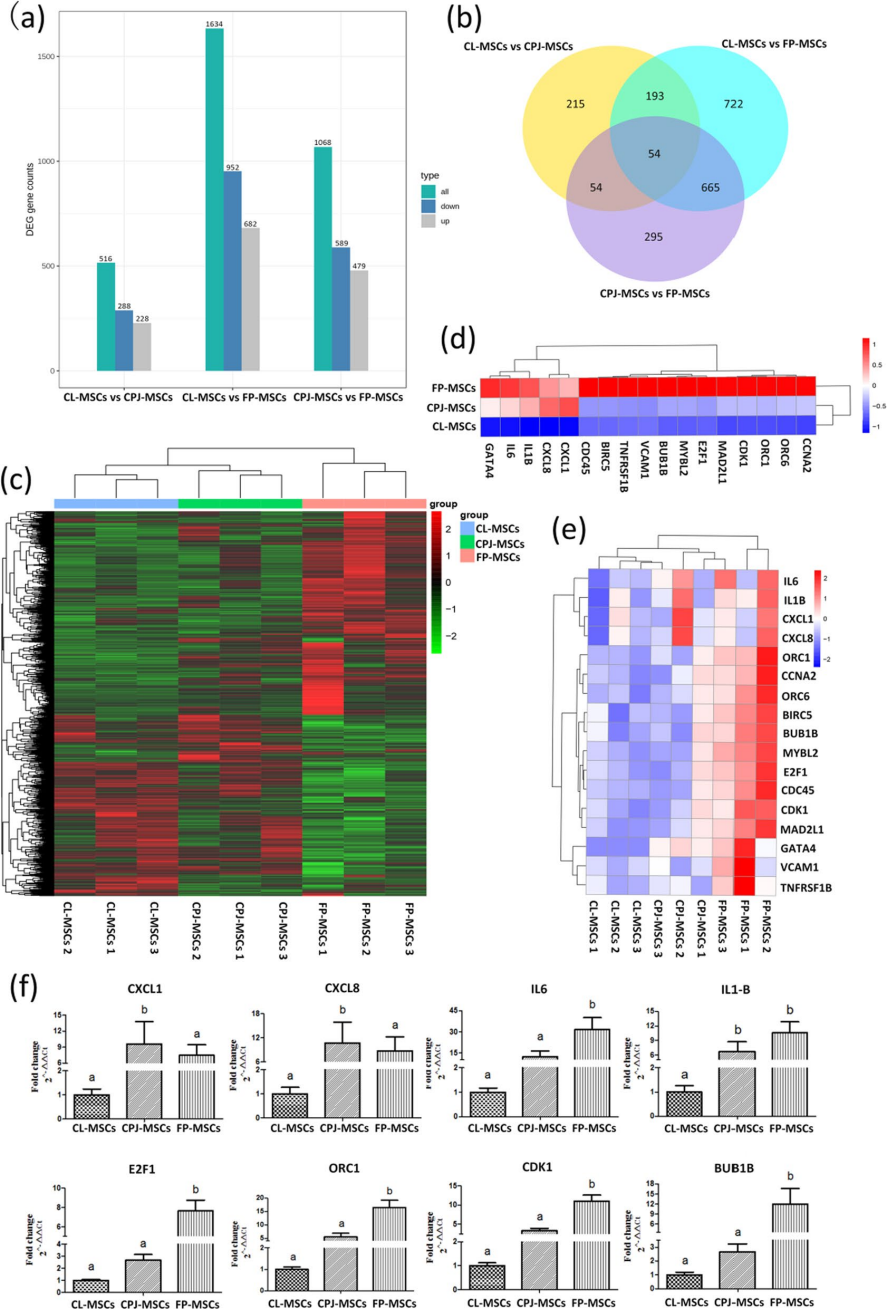
Comparison of heterogeneity of transcriptome and inflammatory factors expression of CL-, CPJ- and FP-MSCs.** a** Differentially expressed genes (DEGs) of three MSCs. **b** Venn diagrams. **c** Heat maps indicating the intensity of the total differentially expressed genes. **d** The heat map shows the intensity of differentially expressed genes related to cycle, inflammatory factors and chemokines in the three groups of MSCs. **e** The heat map shows the intensity of differentially expressed genes associated with cycle, inflammatory factors and chemokines in each MSCs. Red denotes upregulated genes, and blue denotes downregulated genes. **f** The expression of related genes verified by qRT-PCR. Different superscripts within a column indicate a significant difference (*p* < 0.05), *n* = 3 for each type of MSCs

The results of DEGs among CL-, CPJ- and FP-MSCs are summarized in Fig. 2a, as shown in the clustering analysis according to the Venn diagram in Fig. 2b. We performed cluster analysis on the differentially expressed genes of the three types of MSCs, and the heat map presents the 751 DEGs among the MSCs which are listed in Fig. 2c. Among the DEGs, the genes related to inflammation and immune regulation were highly expressed in FP-MSCs and CPJ-MSCs but were low expressed in CL-MSCs. The genes related to cell cycle were highly expressed in FP-MSCs but were low expressed in CL-MSCs. Meanwhile, the expression of these genes in CPJ-MSCs was higher than in CL-MSCs but lower than in FP-MSCs (Fig. 2d, e). The results showed that transcriptomic differences exist among the three types of MSCs derived from perinatal tissues donated by same donors. Particularly, the genes related to inflammation and immune regulation in CL-MSCs were very differentially expressed compared to those in CPJ- and FP-MSCs.

In addition, the expressions levels of genes related to inflammatory factors and immune regulation (CXCL1, CXCL8, IL6 and IL1B) and cell cycle (E2F1, ORC1, CDK1 and BUB1B) in CL-MSCs, CPJ-MSCs and FP-MSCs by qRT-PCR shown in Fig. 2f were consistent with the results of differential gene expression analysis.

### Comparison of immunomodulatory cytokines and chemokine generated by CL-MSCs, CPJ-MSCs and FP-MSCs under the stimulation of IFNγ in vitro

**Fig. 3:**
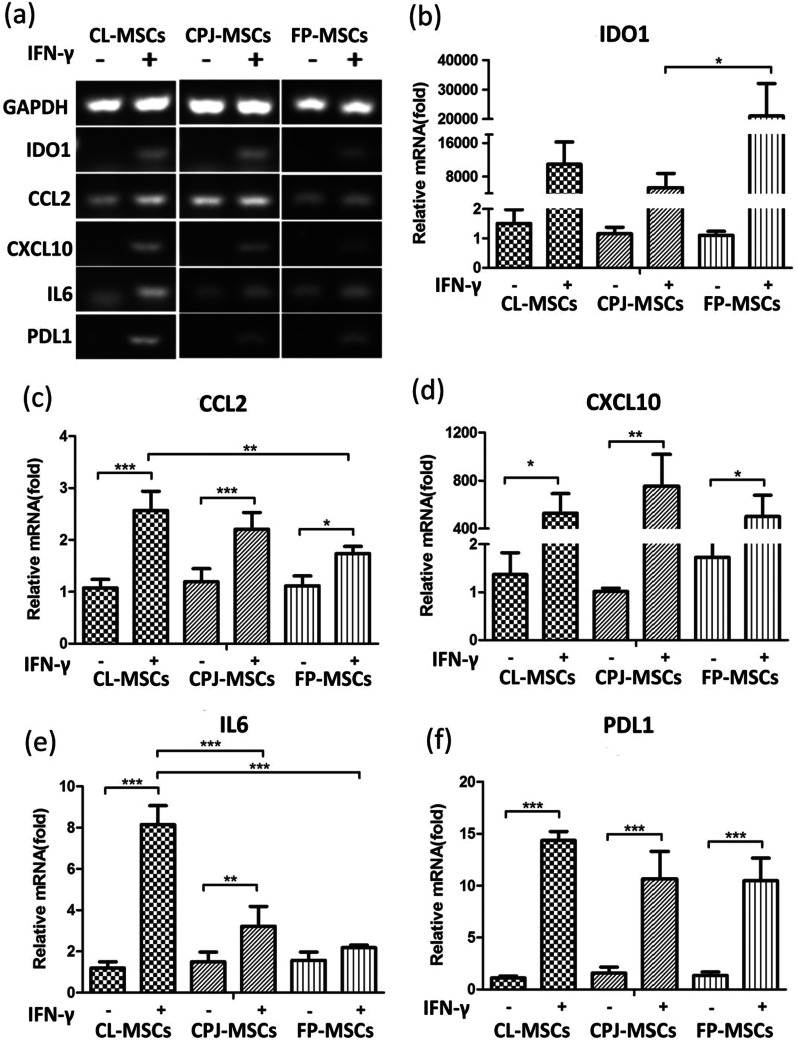
Chemokine and migration-related gene expression of MSCs under the treatment of IFNγ in vitro.** a** Detection of cytokine expression by reverse transcriptase gel electrophoresis. **b, c, d, e, f** Detection of cytokine expression by qRT-PCR, indoleamine 2,3-dioxygenase 1 (IDO1) (b). C–C motif chemokine 2 (CCL2) (c). C-X-C motif chemokine 10 (CXCL10) (d). Interleukin-6 (IL6) (e) and programmed cell death 1 ligand 1 (PDL1) (f). * represents a significant difference (*p* < 0.05). ** represents a significant difference (*p* < 0.01). *** represents a significant difference (*p* < 0.001), and *n* = 3 for each group

It is well known to use pro-inflammatory cytokines such as IFN-γ stimulating MSCs which can change the expression profile of cytokines and enhance the immunosuppressive effect of MSCs [25]. Genes associated with indoleamine 2,3-dioxygenase 1 (IDO1) (Fig. 3a, b), C–C motif chemokine 2 (CCL2) (Fig. 3a, c), C-X-C motif chemokine 10 (CXCL10) (Fig. 3a, d), interleukin-6 (IL6) (Fig. 3a, e), programmed cell death 1 ligand 1(PDL1) (Fig. 3a, f), respectively, upregulated after being treated with IFNγ for 24 h, suggesting that the MSCs may respond to the inflammatory stimuli. Of note, CL-MSCs show significantly higher expression of IL6 and CCL2 than FP-MSCs and CPJ-MSCs (Fig. 3c, e).

### CL-MSCs, CPJ-MSCs and FP-MSCs showed different therapeutic effects on ALF mouse model.

**Fig. 4:**
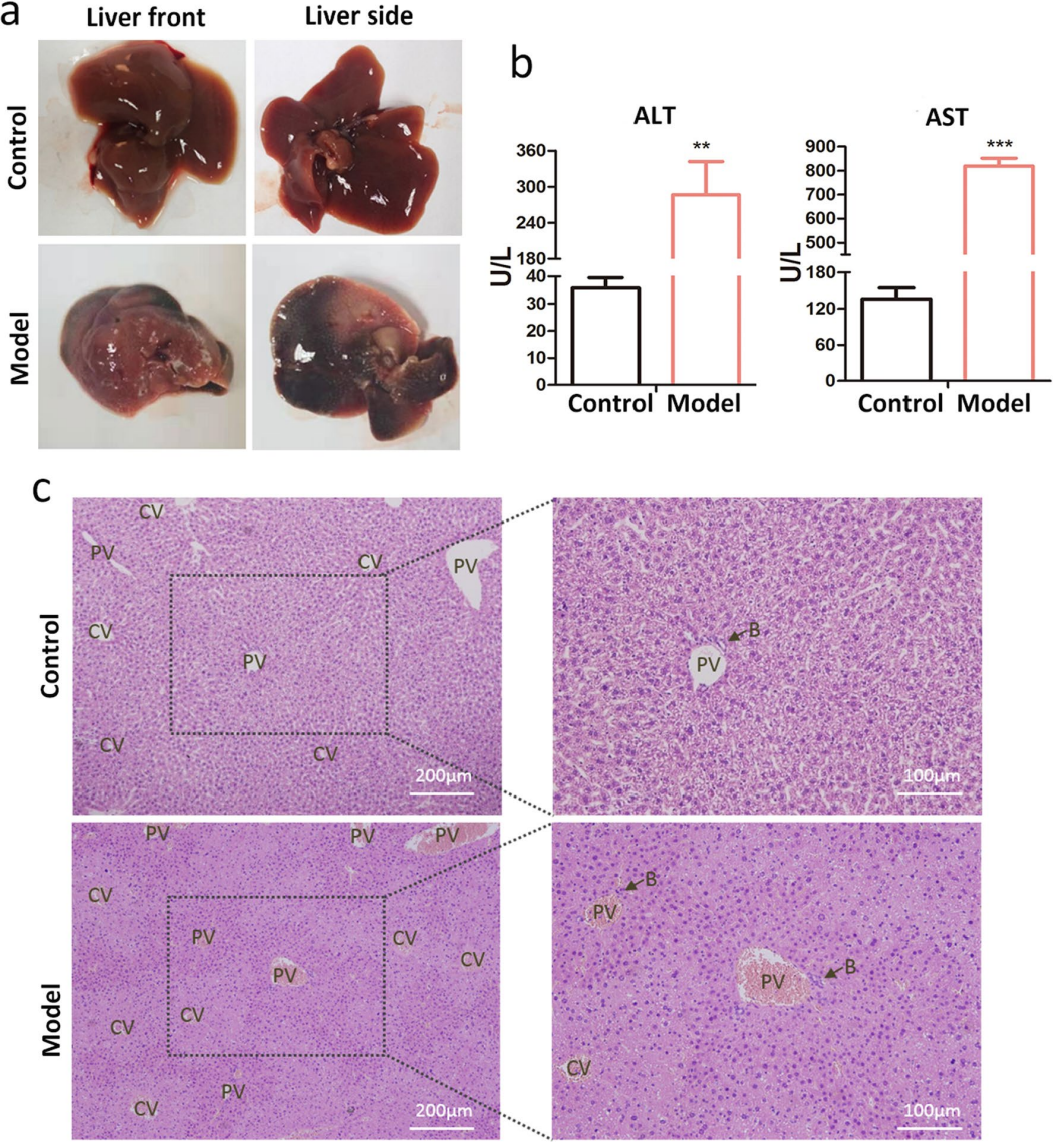
The generation of hepatic failure mouse model.** a** Liver morphological comparison between normal mouse and CCl_4_-administered ALF model. **b** Serum ALT and AST detected by automatic biochemical instrument (*n* = 6). **c** Liver histology after 24 h of CCl_4_ treatment. H&E staining showing hepatic lobule necrosis in model mice but not in normal mice. CV (Central vein), PV (portal vein) and B (bile ducts). * represents a significant difference (*p* < 0.05). ** represents a significant difference (*p* < 0.01). *** represents a significant difference (*p* < 0.001)

**Fig. 5:**
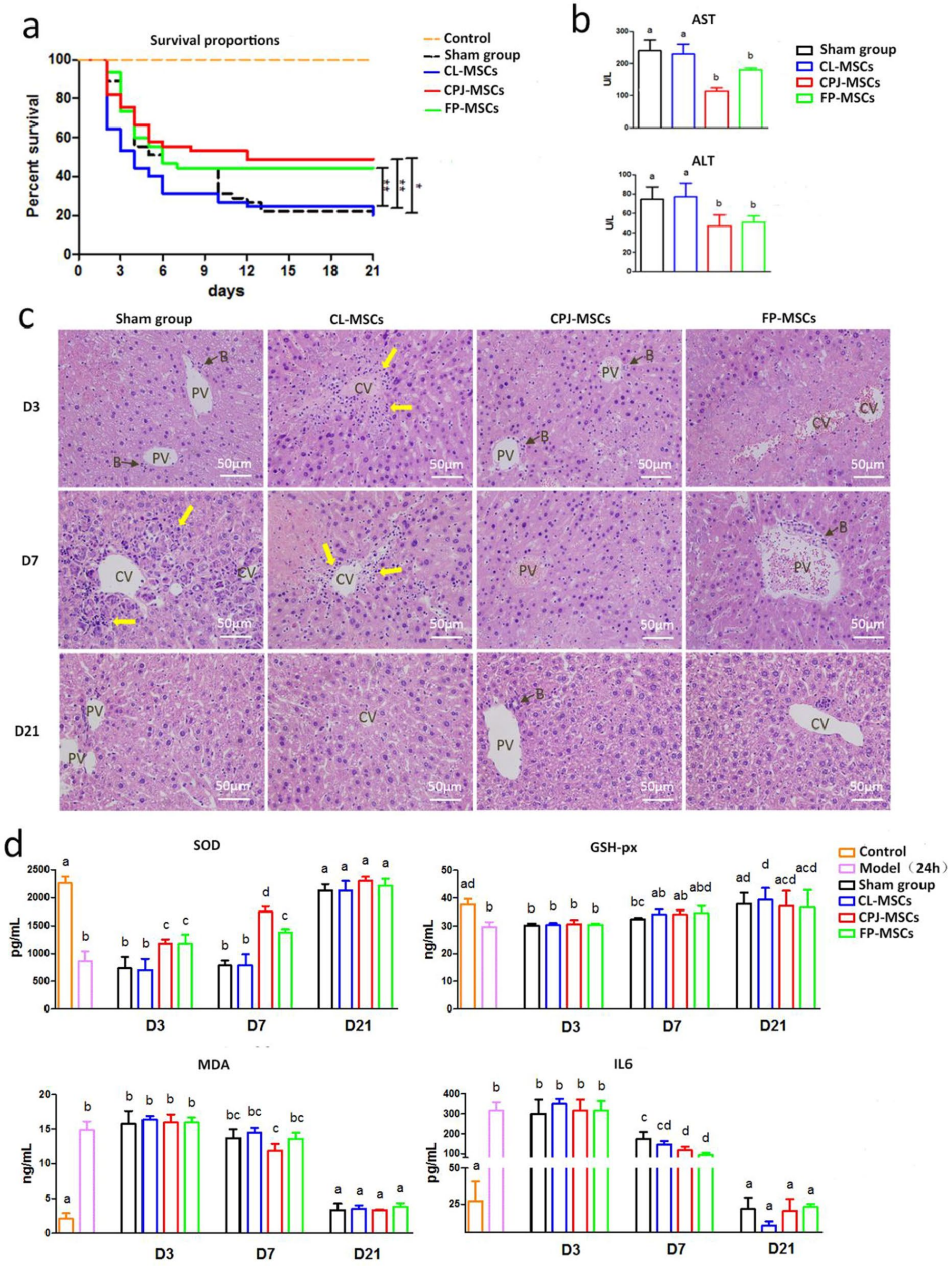
Different therapeutic effects of CL-, CPJ- and FP-MSCs on the treatment of ALF mice. **a** Survival rates of the CCl_4_-induced ALF mouse model treated by different types of MSCs (*n* = 15 for control group and *n* = 45 for other groups). **b** Serum ALT and AST levels (*n* = 6 for each group). **c** H&E staining of liver sections from different treatment groups (400 ×). CV (Central vein), PV (portal vein) and B (bile ducts). **d** SOD, GSH-px, MDA, IL6 in liver homogenate detected by ELISA (*n* = 6 for each group). * represents a significant difference (*p* < 0.05). ** represents a significant difference (*p* < 0.01). *** represents a significant difference (*p* < 0.001). Different superscripts within a column indicate a significant difference (*p* < 0.05)

For the successful generation of the hepatic failure mouse model, livers of CCl_4_-treated mice showing a pale and irregular surface which may be indicative of severe hepatocellular damage at 24 h post-CCl_4_ treatment compared to the liver of normal mice had a smooth surface with uniform and soft textures (Fig. 4a). The ALF mouse model was validated by the massive necrosis in histological examination. After 24 h and 3 days of CCl4 treatment, H&E staining showed hepatocyte necrosis around the central vein (Figs. 4c, 5c). After 7 days of CCl_4_ treatment, H&E staining showed proliferative multinucleated hepatocytes, and Mallory corpuscles appeared around the central vein, accompanied by inflammatory cell infiltration (Fig. 5c). Besides, 24 h post-CCl_4_ treatment, the concentrations of ALT and AST in blood serum increased significantly (Fig. 4b). The levels of SOD and GSH-px decreased significantly, and the level of MDA increased significantly in liver homogenate (Fig. 5d), which indicated that the livers of ALF mouse models were seriously injured.

After the success in generation of ALF model, we subsequently infused three types of MSCs into the ALF mice via tail vein injection. After a 21-day follow-up post-cell transplantation, the survival rates of the ALF mice received intravenous CPJ-MSCs and FP-MSCs therapy significantly improved compared to that of Sham group (*p* < 0.05). Totally, 22 out of 45 and 20 out of 45 ALF mice in CPJ and FP groups survived, respectively, while only 10 out of 45 ALF mice survived in Sham group. However, the CL-MSCs therapy failed to improve the survival of ALF mice (11 out of 45 ALF mice survived), which is similar to that of Sham group (*p* > 0.05) (Fig. 5a). At 7 d post-therapy, the infusion of CPJ-MSCs or FP-MSCs generated significant therapeutic benefit and effects on depression of serum ALT and AST levels than those of CL-MSCs. In particular, CPJ-MSCs showed the best therapeutic effect on depression of the concentration of ALT and AST (Fig. 5b).

The HE staining of liver pathology showed that after 3 d of MSC therapy, inflammatory cells invaded can be observed in CL group, which was not found in CPJ or FP groups. Moreover, after 7 d of infusion of CL-MSCs transplantation, inflammatory cell infiltration still obviously existed in the central vein area (Fig. 5c, Additional file 1: Fig. s2).

In addition, we also collected liver tissues at different time points to detect SOD, GSH-px and MDA in liver homogenate of each group. At 3 d post-therapy, the MDA levels were identical among all groups, which indicated similar degree of initial liver injury caused by CCl_4_, while at 7 d post-therapy, CPJ-MSCs infusion significantly reduced the MDA level compared to the saline group, and the infusion of CPJ-MSCs significantly increased the level of SOD compared to other treatment groups (Fig. 5d). Finally, we compared the inflammation in mice after therapy by detecting IL6. At 7 d post-therapy, CPJ-MSCs and FP-MSCs performed more positive effect on reducing of IL6 than CL-MSCs compared to Sham group (Fig. 5d).

## Discussion

The International Society for Cellular Therapy (ISCT) has stipulated the identification criteria of human MSC, which includes the following three requirements: adherent growth under normal culture conditions; positive for CD73, CD90 and CD105 but negative for CD45, CD34, CD14 or CD11b, CD79a or CD19, HLA-DR; and capable to differentiate into adipocytes, chondrocytes and osteoblasts in vitro [26]. Although mesenchymal stem cells from different sources share these similar characteristics, they also exhibit individual properties. Umbilical cord and placenta belong to perinatal tissues, but the individually derived MSCs show different proliferative abilities and differentiation potential [15]. Study also showed that MSCs from umbilical cord and placental possess different paracrine effects [16]. The heterogeneity of MSCs may lead to different clinical indications. In particular, as far as our knowledge, which type of perinatal tissue-derived MSCs is suitable for the treatment of ALF is unclear.

In a previous study, CL -, WJ -, CPJ - and FP-MSCs-derived MSCs from human perinatal tissues were compared for their colony-forming efficiency and differentiation ability in vitro [15]. In our study, we isolated MSCs from three perinatal tissues (CL, FP and CPJ). We compared the transcriptomic DEGs among the three types of MSCs. Our result found that the DEGs in CL-MSCs were mainly enriched in embryonic development functions by GO enrichment analysis (Additional file 1: Fig. s3, Fig. 4), and the DEGs in FP-MSCs were mainly enriched in cell cycle and cytokine activity (Additional file 1: Fig. s3, Fig. 5). By comparing CPJ-MSCs with FP-MSCs, CPJ-MSCs displayed more characteristics of embryonic development than FP-MSCs (Additional file 1: Fig. s4, Fig. 5). Meanwhile, the transcriptomic results showed that CPJ-MSCs showed better immunomodulatory ability than CL-MSCs. Our study demonstrated that among these three types of MSCs derived from umbilical cord and placenta, CPJ-MSCs present strong immune-regulating potential, and CPJ-MSCs imply proliferation and amplification ability.

The MSCs therapy has shown the great potential to become a promising medical interventions to relief symptom and save ALF patient's life [27]. CCl_4_-induced ALF in mouse is a well-established model to mimic human ALF and has been widely applied to study liver failure and develop therapeutic strategies [28]. Therefore, we employed the mouse model of ALF to test the therapeutic potential of CL-, CPJ- and FP-MSCs derived from the perinatal tissues donated by same donors. Both CPJ-MSCs and FP-MSCs show integrated therapeutic effects on ALF mice evaluated by the survival rate, liver pathology and biomedical indicators. However, no improvement was observed in ALF mice after CL-MSCs transplantation. Previous studies reported that immune cells interact with MSCs in the inflammatory microenvironment: MSCs secrete cytokines to regulate immune cells, which will also be affected by the secretion of inflammatory factors by immune cells [29]. In certain addition, for example, when exposed to low levels of IFN γ and TNF, MSCs promote the infiltration of immune cells and result in inflammation instead of playing the immunosuppressive role [29]. In this study, we found that the mortality of mice infused with CL-MSCs in the early stage (day 3) of treatment was higher than that of other groups. Combined with the pathological results, inflammatory cell infiltration occurred after CL-MSCs infusion on days 3 and 7. Moreover, we found that compared to the Sham group, the ability of CL-MSCs to depress IL6 seems less than those of CPJ-MSCs and FP-MSCs on 7 d post-therapy. Simultaneously, the immune response of the three types of MSCs to IFN-γ in vitro was different, and the immunomodulatory cytokines and chemokines secreted by these MSCs were different, among which IL6 and CCL2 were significantly different. The results indicated that CL-MSCs secreted more inflammatory (pro-inflammatory) factors and chemokines under inflammatory environment. The subsequent cell transplantation proved that CPJ-MSCs and FP-MSCs showed efficient therapeutic effects on the ALF mice, but CL-MSC failed to relieve symptom and improve survival rate of ALF mice. Therefore, CPJ-MSCs and FP-MSCs are highly recommended as the most potential seed cells for the treatment of ALF, and the aggravated inflammatory response may be the explanation of the infusion of CL-MSCs leading to the low survival rate of ALF mice.

## Conclusions

In this study, three different types of MSCs (CL-MSCs, FP-MSCs and CPJ-MSCs) were isolated and identified from perinatal tissues donated by same donors. The gene expression profiles and in vitro immune regulation ability and the therapeutic effects of CL-MSCs, FP-MSCs and CPJ-MSCs on ALF mouse model were compared. The results showed that significant transcriptomic differences exist among different types of MSCs. Furthermore, the transplantation of CPJ-MSCs and FP-MSCs improved the survival rate of ALF mice and liver function evaluated by HE staining and liver functional biomedical indexes. However, CL-MSCs failed to treat ALF mice either in symptom or survival rate. Therefore, FP- and CPJ-MSCs are the preferred MSCs for the treatment of ALF.

## Supplementary Information

**Additional file 1: Figure s1.** Surface marker expression of three types of MSCs. **Figure s2.** H & E staining of liver sections from different treatment groups. **Figure s3.** The enrichment analysis of Gene Ontology (GO) between FP-MSCs and CL-MSCs. **Figure s4.** The enrichment analysis of Gene Ontology (GO) between CPJ-MSCs and CL-MSCs. **Figure s5.** The enrichment analysis of Gene Ontology (GO) between FP-MSCs and CPJ-MSCs.

## Data Availability

The obtained transcriptome data have been uploaded into the NCBI SRA database and are accessible via the accession number PRJNA836369.

## Abbreviations

MSCs: Mesenchymal stem cells
CL: Cord lining
CPJ: Cord–placenta junction
FP: Fetal placenta
ALF: Acute liver failure
CCl_4_: Carbon tetrachloride
PFA: Paraformaldehyde
DEG: Differential expression gene
IFNγ: Interferon γ
IDO1: Indoleamine 2,3-dioxygenase 1
CCL2: C–C motif chemokine 2
CXCL10: C-X-C motif chemokine 10, IL6: Interleukin-6
PDL1: Programmed cell death 1 ligand
IACUC: Institutional Animal Care and Use Committees
H&E: Hematoxylin and eosin
AST: Aspartate aminotransferase
ALT: Alanine aminotransferase
SOD: Superoxide dismutase
GSH-px: Glutathione peroxidase
MDA: Malondialdehyde
ISCT: International Society for Cellular Therapy
