# Supplementary material for: Mesenchymal stem cells derived from different perinatal tissues donated by same donors manifest variant performance on the acute liver failure model in mouse
Source: Stem Cell Res Ther. 2022 Jun 3;13:231. doi: 10.1186/s13287-022-02909-w (PMC9166497; doi:10.1186/s13287-022-02909-w)
Supplement: Supplementary file 1 — Additional file 1: Figure s1. Surface marker expression of three types of MSCs. Figure s2. H & E staining of liver sections from different treatment groups. Figure s3. The enrichment analysis of Gene Ontology (GO) between FP-MSCs and CL-MSCs. Figure s4. The enrichment analysis of Gene Ontology (GO) between CPJ-MSCs and CL-MSCs. Figure s5. The enrichment analysis of Gene Ontology (GO) between FP-MSCs and CPJ-MSCs. [file 13287_2022_2909_MOESM1_ESM.pdf]

Figure. s1 Surface marker expression of three types of MSCs.

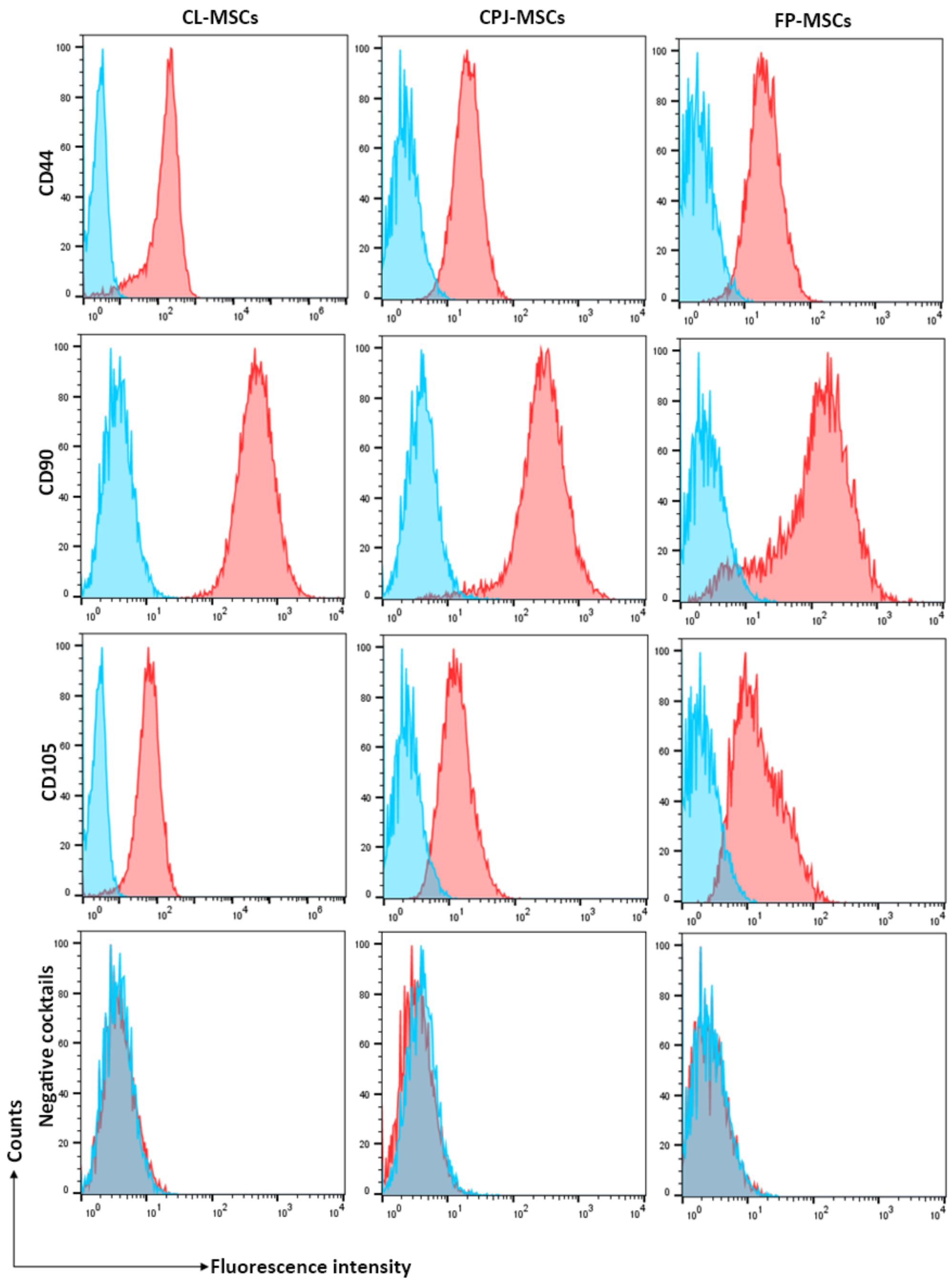

The expression of surface markers of CL-MSCs, CPJ-MSCs or FP-MSCs on CD44, CD90, CD105 and negative cocktails was detected by flow cytometry.

Figure. s2 H & E staining of liver sections from different treatment groups (100×, 200×).

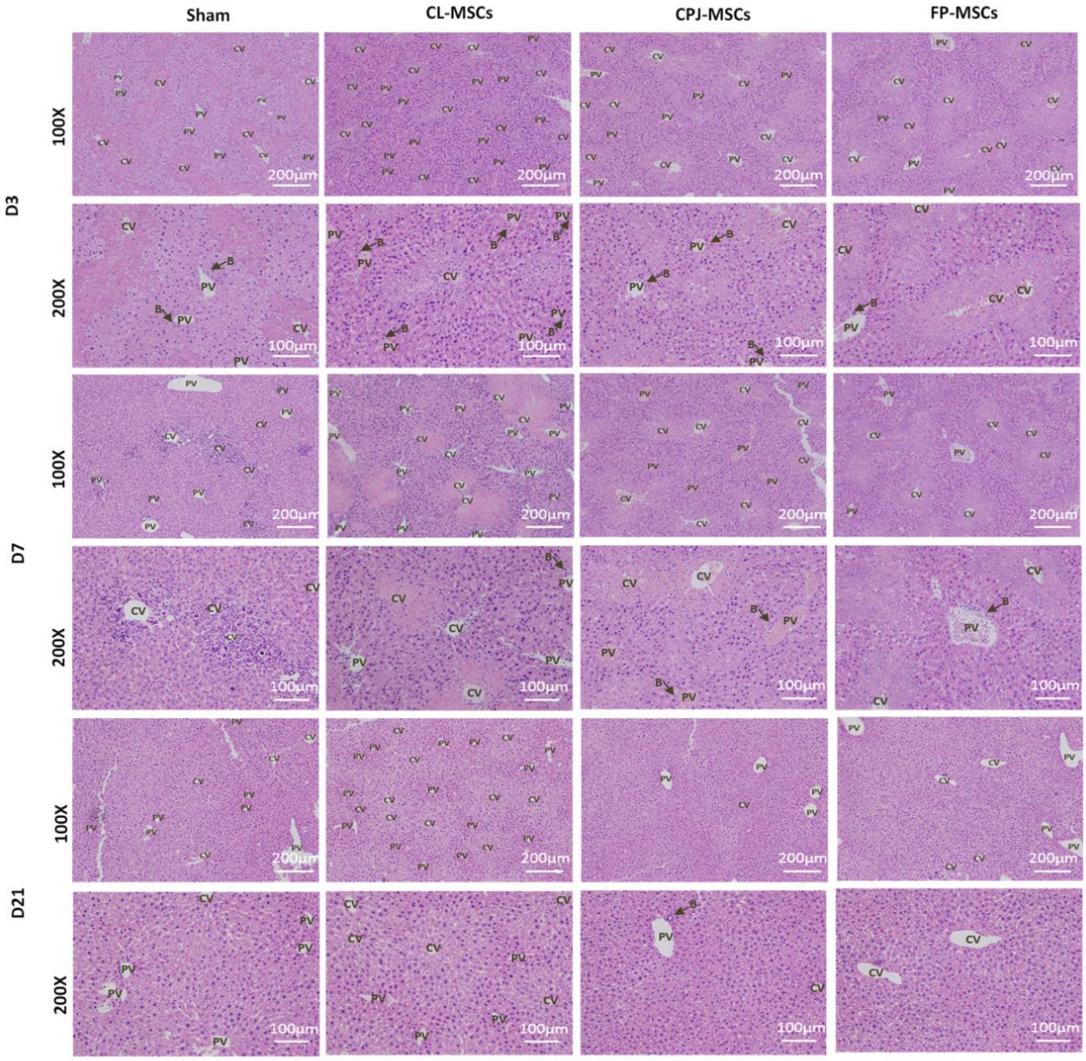

H & E staining of liver sections at 3, 7 and 21 days in different treatment groups (100 ×, 200×). CV, Central vein. PV, portal vein and B, bile ducts.

Figure. s3 The enrichment analysis of Gene Ontology (GO) between FP-MSCs and CL-MSCs

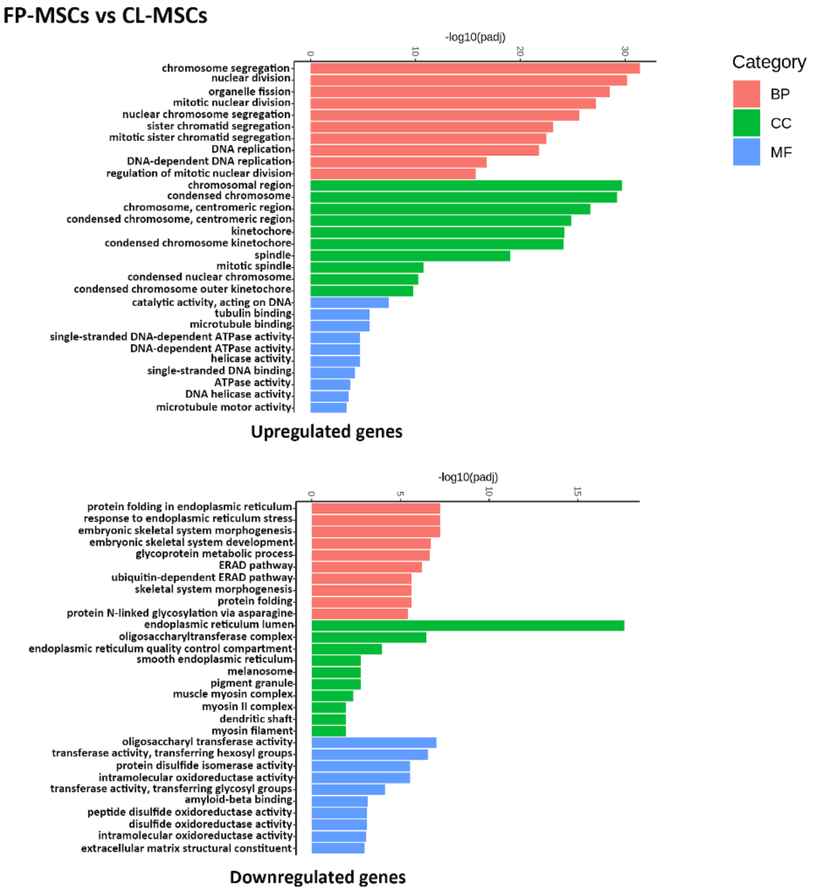

Gene Ontology (GO) enrichment analysis of up-regulated genes and down-regulated genes was compared between FP-MSCs and CL-MSCs. Upregulated genes: highly expressed genes in FP-MSCs. Downregulated genes: highly expressed genes in CL-MSCs. BP, biological process. MF, molecular function. CC, cellular component.

Figure. s4 The enrichment analysis of Gene Ontology (GO) between CPJ-MSCs and CL-MSCs

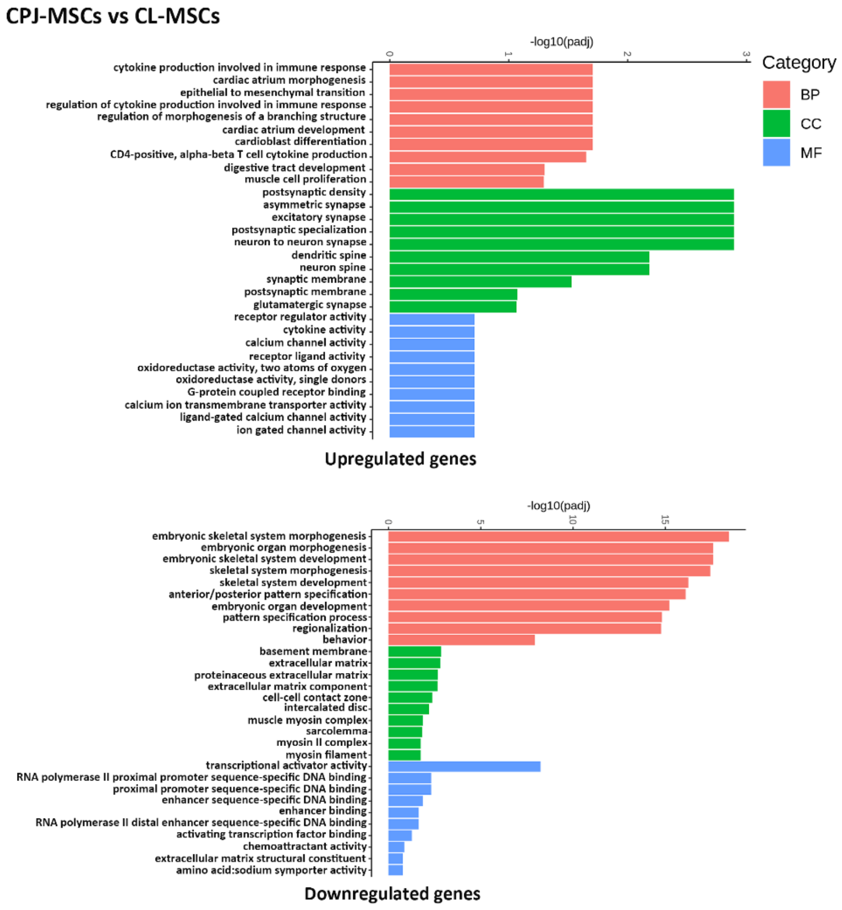

Gene Ontology (GO) enrichment analysis of up-regulated genes and down-regulated genes was compared between CPJ-MSCs and CL-MSCs. Upregulated genes: highly expressed genes in CPJ-MSCs. Downregulated genes: highly expressed genes in CL-MSCs. BP, biological process. MF, molecular function. CC, cellular component.

Figure. s5 The enrichment analysis of Gene Ontology (GO) between FP-MSCs and CPJ-MSCs

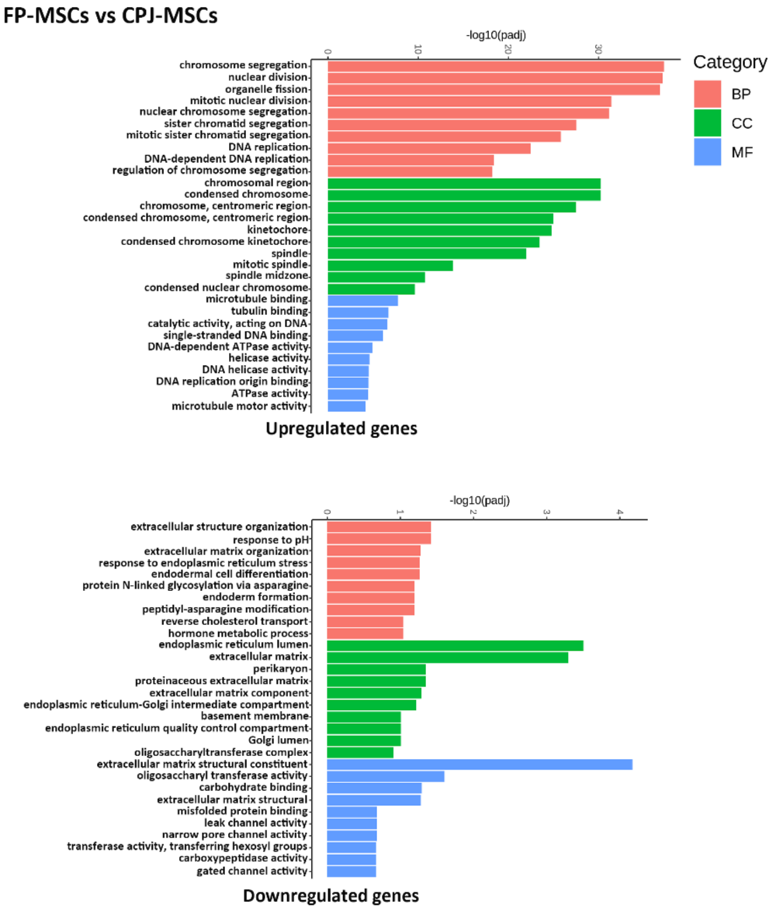

Gene Ontology (GO) enrichment analysis of up-regulated genes and down-regulated genes was compared between FP-MSCs and CPJ-MSCs. Upregulated genes: highly expressed genes in FP-MSCs. Downregulated genes: highly expressed genes in CPJ-MSCs. BP, biological process. MF, molecular function. CC, cellular component.
